# Efficacy of an Objective Structured Clinical Examination Training Approach for Training Pharmacy Students in Diabetes Mellitus Counseling: A Randomized Controlled Trial

**DOI:** 10.3390/pharmacy8040229

**Published:** 2020-11-26

**Authors:** Imaneh Farahani, Samieh Farahani, Maira A. Deters, Holger Schwender, Stephanie Laeer

**Affiliations:** 1 Institute of Clinical Pharmacy and Pharmacotherapy, Heinrich Heine University Duesseldorf, Universitaetsstrasse 1, 40225 Duesseldorf, Germany; samieh.farahani@hhu.de (S.F.); maira.deters@hhu.de (M.A.D.); stephanie.laeer@hhu.de (S.L.); 2 Mathematical Institute, Heinrich Heine University Duesseldorf, Universitaetsstrasse 1, 40225 Duesseldorf, Germany; holger.schwender@hhu.de

**Keywords:** pharmacy education, pharmacy students, OSCE, diabetes mellitus, counseling

## Abstract

Pharmacists’ tasks are multifaceted and include, for example, vital counseling and communication skills. Objective Structured Clinical Examinations (OSCEs) could be used to train pharmacy students in these skills. Our study sought to determine the efficacy of our OSCE training approach for training pharmacy students’ counseling and communication skills on diabetes mellitus compared to a control group. This randomized controlled study was conducted with pharmacy students using a pre-post-design. The intervention group completed diabetes OSCE training, while the control group solved diabetes patient cases using subjective, objective, assessment, and plan notes. Before and after the respective training, both groups completed OSCEs evaluating counseling and communication skills. Before each OSCE encounter, the participants completed a self-assessment questionnaire and, upon completion of the seminar, filled out a satisfaction survey. The OSCE-trained group demonstrated a significantly greater increase in counseling and communication skills and self-confidence than the control group. Both groups were generally satisfied with the seminar. These results demonstrate that our OSCE training approach allows for the effective training of pharmacy students’ diabetes counseling and communication skills and suggests the inclusion of such a skill-based approach more widely in pharmacy students’ education.

## 1. Introduction

Pharmacists are responsible for supplying patients and health care professionals with medicines and other health care products and counseling them concerning their proper usage. Along with conveying the importance of following the correct medical regimen, pharmacists should ensure that patients are aware of the correct timing of doses, drug–drug interactions, and drug–food interactions, as well as possible adverse medicine events. Additionally, patients’ adherence should be supported [1]. Community pharmacists, as accessible health care professionals and experts in drug therapy, are well-positioned to contribute to patients’ adherence to long-term therapy [2,3].

Globally, the majority of pharmacists work in community pharmacies [4]. For example, approximately 80% of pharmacists in Germany [5] and 65% of pharmacists in Canada [6] work as community pharmacists. Consequently, pharmacists must be able to provide optimal counseling to contribute properly to their patients’ therapy.

“Adherence” and “correct application” are crucial issues for patients with diabetes mellitus [7]. In 2019, there were 463 million diabetes mellitus patients worldwide [8]. Poor adherence is common among patients with diabetes mellitus and is associated with poor glycemic control, increased risk of hospitalization, increased mortality, and higher costs [7,9,10,11]. Several investigations have shown the benefits of involving pharmacists in the therapy management of diabetes mellitus patients [12,13]. Proper medication counseling is important in enhancing patients’ adherence [14,15]. Therefore, promoting counseling and communication skills in pharmacy students is essential to the fulfillment of their future role as community pharmacists.

A multifaceted approach is required to address the complexity of clinical education [16], including the teaching of counseling and communication skills. A possible way to address these complexities may be the use of Objective Structured Clinical Examinations (OSCEs). OSCEs are defined as “an approach to the assessment of clinical competence in which the components of competence are assessed in a planned or structured way with attention being paid to the objectivity of the examination” [17]. OSCEs can be formative when used as a learning tool or summative when used as an evaluation of clinical skills or knowledge [18]. OSCEs provide a safe environment for students to apply clinical skills without risk to patients [19].

The use of OSCEs as a learning tool has been described in different settings, such as under examination-like conditions with additional feedback [18,20] or more extensive training conditions [21,22,23] However, their effectiveness is controversial [18,20,24,25]. Gums et al. found a significant improvement in OSCE performance after an individualized formative assessment in a laboratory session [25], which can be considered as a formative OSCE-like approach. However, Chisnall et al. reported that formative OSCEs did not result in a significant change in the overall pass rate of summative OSCEs, but found improved performance in subsequent summative OSCEs only in particular stations [18]. Alkhateeb et al. found that formative OSCEs did not result in a significant difference in pass rate and that the group without formative OSCEs achieved even higher OSCE scores [20]. Nevertheless, OSCEs as a learning tool are well-received by students [21,23,26] but are facility-, time-, cost-, and personnel-intense [18,23,27]. Group-based OSCEs (GOSCEs) [23,28] or peer assessed OSCEs [29] (POSCEs) may address some of the problems encountered with using OSCEs as a learning tool. In GOSCEs, the learners rotate in groups around the stations rather than as individuals, and learners can observe each other executing the clinical task at each station [30,31]. POSCEs allow students to gain OSCE experience and are well-received by both the assessed and assessors [26,32].

This study investigated the efficacy of our OSCE training approach for training German pharmacy students in counseling on diabetes mellitus compared to a control group.

## 2. Materials and Methods

### 2.1. Operational Definitions

For the purpose of this article, we used the term “formative OSCEs” to refer to OSCEs for training purposes used for the intervention group in this study. The term “patient cases” refers to the training of the control group in which patient cases were solved by the preparation of subjective, objective, assessment, and plan (SOAP) notes. For the purpose of this study, the term “summative OSCEs” was used to refer to the assessment of the participants’ performance twice during the study. The summative pre-training OSCEs assessed the participants’ basic performance prior to training, while summative post-training OSCEs assessed the participants’ final performance.

### 2.2. Participants and Study Design

This study assessed the effect of our OSCE training approach using a randomized controlled trial with a pre-post-design. The investigation was conducted in the April–June 2019 period during the clinical pharmacy course at Heinrich Heine University Duesseldorf. The language of the investigation was German. All data were collected in pseudonymous form, with the exception of an anonymous satisfaction survey. All data were rendered anonymous following analysis. Approval for this study was granted by the responsible ethics committee (Number 2019-467-ProspDEuA).

**Figure 1:**
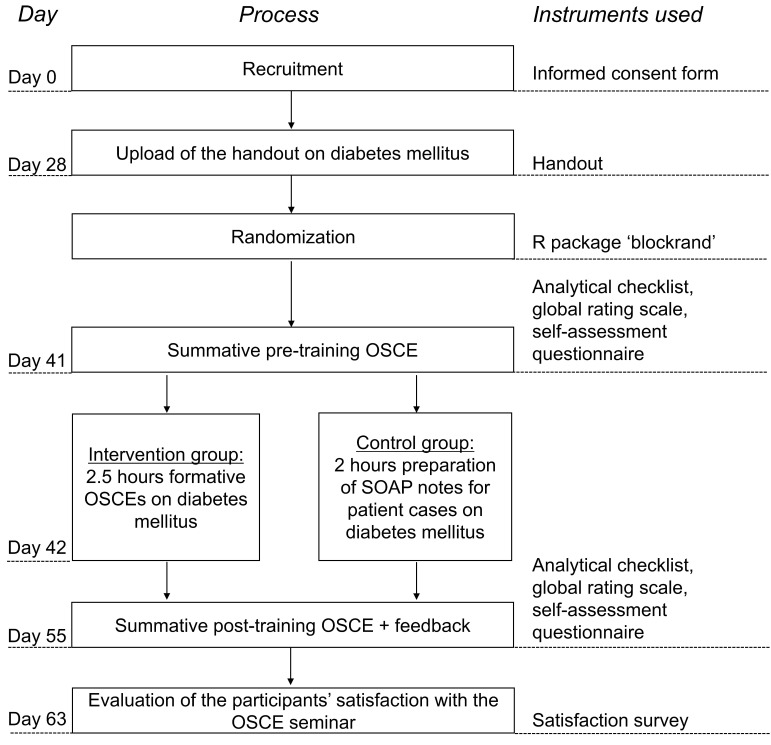
Overview of the study design. OSCE = Objective Structured Clinical Examination; SOAP = Subjective, Objective, Assessment, Plan.

Fifty-eight students in the eighth and final semester of their university pharmacy studies were invited to participate in the study in April 2019. Participants who signed the informed consent form were randomized to either intervention group or control group using the R [33] package “blockrand”. The study design is illustrated in Figure 1.

### 2.3. Seminar Procedure

At the beginning of the seminar, a diabetes mellitus handout was uploaded online. After about two weeks, the participants completed a summative pre-training OSCE and the first multiple-choice test on diabetes mellitus on the same day. The next day, the participants completed training depending on their group. Participants in the intervention group attended an OSCE training approach (with formative OSCEs) for 2.5 h, while the control group was trained using the university’s traditional teaching method, involving the preparation of SOAP notes for 2 h to solve diabetes mellitus patient cases. Two weeks after the training, all participants completed a summative post-training OSCE and a second multiple-choice test on diabetes mellitus. One week after the summative post-training OSCE, the participants’ satisfaction with the OSCE seminar was surveyed.

### 2.4. Instruments

#### 2.4.1. Handout

A 24-page (without references) diabetes mellitus handout covering general information, therapy, and complications of diabetes mellitus based on national guidelines [34,35] was prepared by a pharmacist and reviewed by another pharmacist. The handout, aiming to bring the participants’ knowledge on diabetes mellitus to the same level, was uploaded online two weeks before the summative pre-training OSCE and was accessible to all eighth-semester pharmacy students throughout the whole semester.

#### 2.4.2. OSCE Cases

The pharmacist who prepared the handout generated 12 OSCE cases on diabetes mellitus type 2 with hypertension and/or dyslipidemia comorbidities (Appendix A), which were reviewed by the pharmacist who reviewed the handout. Half of the OSCE cases dealt with the introduction of an antidiabetic drug (initiation of therapy) while the other six cases dealt with a follow-up prescription of an antidiabetic drug (implementation of therapy). The OSCE cases were designed to be completed within a maximum of 10 min. Six OSCE cases were used in the summative pre-training OSCE while the remaining cases were used in the summative post-training OSCE. The cases used for the summative pre-training OSCE were reused for the training OSCEs in the intervention group.

#### 2.4.3. Scoring Instruments

An analytical checklist and a global rating scale were used to evaluate the participants’ performance. An observer filled out the analytical checklist for each participant to assess changes in counseling performance between summative pre- and post-training OSCEs. The global analytical checklist for OSCEs, adopted from other studies [22,36], was adjusted for each OSCE case and focused on the content and structure of the counseling. Consequently, 12 OSCE case-specific analytical checklists were created, with varying total scores; therefore, the analysis was carried out in percentages or percentage points. Supplemental Materials 1 and 2 show two examples of the translated case-specific checklists. The checklists included exemplary dialogues to facilitate the observers’ task. One point was given when the participant addressed the respective item correctly; if not, zero points were awarded. The analytical checklists comprised the following sections: “greeting”, “medical history”, “drug information” (initiation or implementation), “prevention”, “goal setting”, “patient involvement”, and “risk communication”. To complete the OSCE case correctly, the participants had to ask the correct questions to receive vital patient information and to give appropriate advice, detect or prevent drug-related problems, and, where applicable, clarify the patient’s questions. A global rating scale for OSCEs, also used in other studies [36], was modified beforehand [22] and applied to assess the participants’ communication skills during the summative pre- and post-training OSCEs. A six-point Likert scale awarded scores from 0 (poor behavior) to 5 points (optimal behavior). The global rating scale focused on the sections “verbal communication skills”, “non-verbal communication skills”, and “patient-centered communication”.

#### 2.4.4. Multiple-Choice Test on Diabetes Mellitus

Different multiple-choice tests on diabetes mellitus assessed the participants’ knowledge immediately after the summative pre- and post-training OSCEs, each consisting of four questions. Supplemental Materials 3 and 4 show the translated multiple-choice tests. The test was completed in the same lecture hall as the summative OSCEs.

#### 2.4.5. Self-Assessment Questionnaire

Participants filled out a self-assessment questionnaire, modified from the one used in PharmAdhere [36], before each summative OSCE to record their self-assessment of their proficiency and confidence regarding their counseling skills before and after the respective training. In Supplemental Material 5, the translated self-assessment questionnaire is depicted. The assessment used a six-point Likert scale from 0 (strongly disagree) to 5 points (strongly agree). The pre-training questionnaire also collected the participants’ demographic characteristics including age, gender, additional education as pharmaceutical technical assistants, and current or former work counseling patients in a community pharmacy.

#### 2.4.6. Participants’ Satisfaction Survey

Participants completed a survey to assess their satisfaction with the seminar. The questionnaire comprised 10 items and used a six-point Likert scale from “strongly disagree” to “strongly agree”. Additional free-text items asked what they favored most and what they would suggest changing. For analysis, the comments on the free-text items were categorized into themes.

#### 2.4.7. Preparation Questionnaire

Participants completed a survey after each summative OSCE to determine their preparation, which inquired as to whether they had prepared for the particular summative OSCE and, if yes, the duration of and tools used for preparation.

### 2.5. Summative OSCEs

Participants completed a summative pre-training OSCE and two weeks later a summative post-training OSCE. The summative OSCEs comprised one station which simulated a patient encounter. A standardized patient, an observer, and a participant attended each OSCE encounter. The participant’s task was to take over the role of the pharmacist and counsel the patient on the use of an antidiabetic drug and solve or prevent potential drug-related problems. Each OSCE case began with a one-minute pre-encounter phase, in which the participant could read the short instruction and the product characteristics of the antidiabetic medication the case dealt with. After the pre-encounter period, a ten-minute encounter period began with the standardized patient handing over a prescription on an antidiabetic drug to the participant. If in the course of the counseling the participant found out that the patient’s medication includes in addition other drugs than the drug on the prescription, the other respective product characteristics were provided. Performance in the OSCE was assessed by the observer using the case-specific analytical checklist and global rating scale. Three pharmacists experienced in rating OSCEs performed the role of observers, allowing three simultaneous patient encounters regarding three different OSCE cases from a pool of six to occur in a single lecture hall The summative post-training OSCEs used a different pool of six cases. The observers received instructions for filling out the analytical checklist and global rating scale. The standardized patients were portrayed by pharmacists (faculty members) or pharmacy students in the eighth semester who were not participants. The standardized patients read their script and received additional instructions prior to the OSCEs. Following the completion of the summative post-training OSCEs, participants received additional feedback on their performance from the observer immediately after the patient encounter.

### 2.6. Training for the Intervention Group

Training for the intervention group consisted of a short lecture on structured pharmaceutical counseling based on the analytical checklist and formative OSCEs with peer assessment. During the training OSCEs, the participants practiced, in groups of four to five, the OSCE case which they had to complete in their summative pre-training OSCEs. Consequently, each of the six groups trained on a different OSCE case. In each group, one member functioned as the pharmacist, one as the standardized patient, and the remaining members as observers, taking turns in each role. The participants used the global analytical checklist which was not case-specific to standardize their assessment and feedback from the observers. The checklists were only provided to the intervention group during the 2.5 h training and were returned at the end of that training. Additionally, the lecture slides were not made available. After practicing the OSCE cases in groups, two participants from each group, with one portraying the pharmacist and the other the patient, presented their practiced patient counseling to the other groups and two trainers. The trainers completed the checklists and global rating scale. Following the presentation, the presenters received feedback from the other groups and the trainers.

### 2.7. Training for the Control Group

The five diabetes mellitus patient cases used for the training of the control group were designed and reviewed by the pharmacists involved in developing the handout and OSCE cases. Medications and problems used in the summative pre-training OSCE were integrated into the patient cases. Appendix B shows a short description of the patient cases. The students were divided into 10 groups of three or four participants, with each group assigned one of the five patient cases. The participants prepared SOAP notes and presented and discussed their solutions with the other groups and a trainer (a pharmacist). The students who did not sign the informed consent form took part in the control training without their data being collected.

### 2.8. Statistical Methods

Scores are reported in percentages or percentage points to permit comparison between the different OSCE cases. *p*-values were calculated for the analytical checklist score, global rating scale score, and self-assessment score. We used a one-sided paired two-sample Wilcoxon test with a significance level of alpha = 0.05 to analyze the increase in the respective score from pre-training to post-training for each group. We assessed whether the increase in the respective score was higher in the intervention group than in the control group using a one-sided Mann–Whitney test with a significance level of alpha = 0.05. A two-sided Mann–Whitney test with a significance level of alpha = 0.05 was used to compare the baseline scores between the groups. Asymptotic p-values are reported. *p*-values were not adjusted for multiple testing. Microsoft Excel 2019 [37] was used for data entry and Origin 2019 [38] was used for analysis.

## 3. Results

### 3.1. Participants

Of the 58 available eighth-semester pharmacy students invited for participation, 52 signed the informed consent form. From these, three were excluded from the analysis due to the non-attendance of the summative OSCEs or the training day. Of the six non-participating students, three assisted voluntarily as standardized patients, and three participated in the control group without their data being collected. Overall, complete data were collected from 49 participants. The demographic characteristics of the intervention and the control group are described in Table 1.

### 3.2. Analytical Checklists for OSCEs

**Figure 2:**
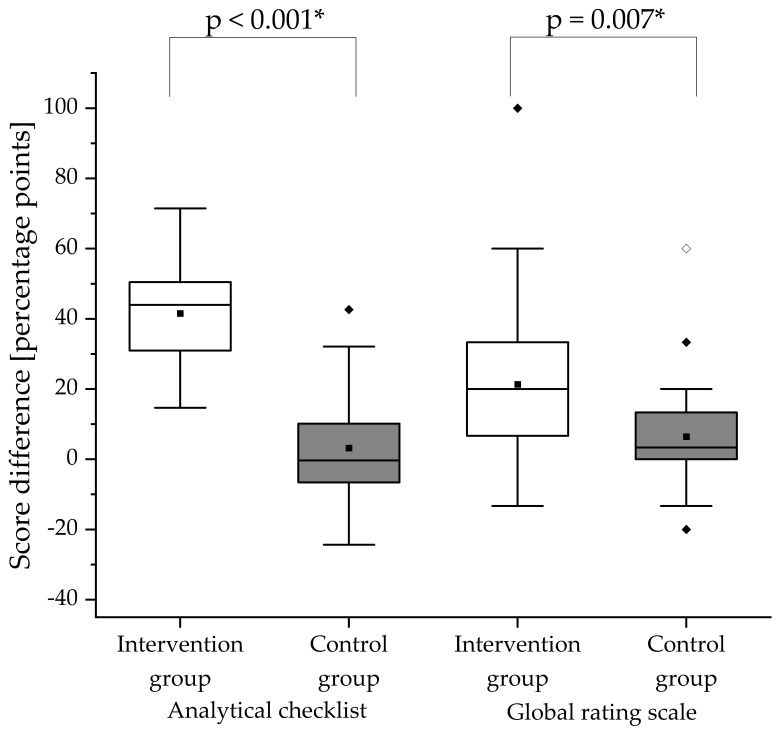
Score differences in the analytical checklist and global rating scale between summative pre- and post-training OSCEs. The difference of each score was obtained by calculating post-training score minus pre-training score for the respective group. White boxes display the intervention group and gray boxes the control group. The black square (■) indicates the mean and the line indicates the median. Outliers are indicated by black diamonds (♦) and extreme values by white diamonds (♢). The asterisk (*) indicates a significant difference. A significance level of alpha = 0.05 was used. N = 25 in the intervention group and n = 24 in the control group.

The participants’ counseling skills were assessed in the summative pre- and post-training OSCEs using analytical checklists. At baseline (pre-training OSCE), the analytical checklist scores did not differ significantly between the intervention and control group (*p* = 0.322). In Figure 2, the change in counseling performance is depicted. The intervention group demonstrated a significant improvement in counseling skills from the summative pre- to post-training OSCE (*p* < 0.001). In contrast, the control group showed no significant improvement (*p* = 0.242). The intervention group showed a significantly greater increase in the analytical checklist score between the pre- and post-training OSCE than the control group (*p* < 0.001). Table 2 shows the scores achieved in the analytical checklist for each section of the analytical checklist per group.

### 3.3. Global Rating Scale

The participants’ communication skills were assessed in the summative pre- and post-training OSCEs using a global rating scale. At baseline (pre-training OSCE), the global rating scale scores did not differ significantly between the intervention and control group (*p* = 0.172). In Figure 2, the change in communication skills is illustrated. While the communication skills of the intervention group improved significantly from the pre- to post-training OSCE (*p* < 0.001), the improvement in the control group was not significant (*p* = 0.066). The intervention group showed significantly higher improvement of communication skills (*p* = 0.007) than the control group. Table 3 shows the scores in percentage or percentage points achieved per section of the global rating scale.

### 3.4. Multiple-Choice Test on Diabetes Mellitus

The participants showed a decline in knowledge scores in both the intervention and control groups from the first multiple-choice test to the second multiple-choice test (Table 4).

### 3.5. Self-Assessment Questionnaire

**Figure 3:**
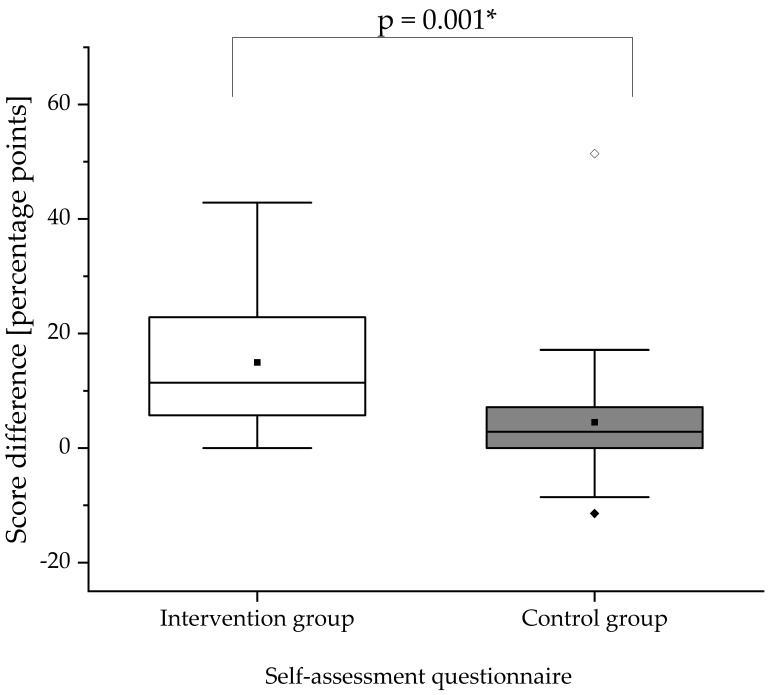
Score differences in the self-assessment questionnaire between summative pre- and post-training OSCEs. The difference of the score was obtained by calculating post-training score minus pre-training score for the respective group. The white box displays the intervention group and the gray box the control group. The black square (■) indicates the mean and the line indicates the median. Outliers are indicated by black diamonds (♦) and extreme values by white diamonds (♢). The asterisk (*) indicates a significant difference. A significance level of alpha = 0.05 was used. N = 25 in the intervention group and n = 24 in the control group.

In Figure 3, the change in self-assessment score is illustrated. At baseline (pre-training OSCE), the self-assessment scores did not differ significantly between the intervention and control group (*p* = 0.157). The self-assessment scores for both groups significantly increased from summative pre- to post-training OSCE (intervention group: *p* < 0.001; control group: *p* = 0.038). The increase in self-assessment score was significantly higher in the intervention group compared to the control group (*p* = 0.001). The increase in the participants’ self-assessment score implies an improvement of self-confidence. Scores achieved in the analytical checklist, global rating scale, and self-assessment questionnaire are documented in Appendix C.

### 3.6. Participants’ Satisfaction

**Figure 4:**
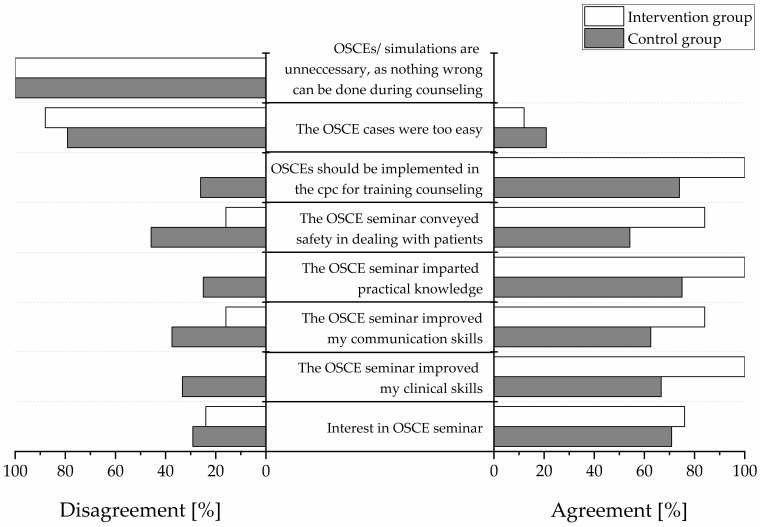
Participants’ satisfaction in percentage. “Agreement” indicates positive responses (“strongly agree”, “agree”, “slightly agree”) “Disagreement” indicates negative responses (“slightly disagree”, “disagree”, “strongly disagree”). White bars display the intervention group and gray bars the control group. N = 25 in the intervention group and n = 24 in the control group. OSCE = Objective Structured Clinical Examination. cpc = clinical pharmacy course.

All participants completed a satisfaction survey (Figure 4). Responses regarding free-text items are depicted in Table 5. The responses were simplified as “agreement” (“strongly agree”, “agree”, and “slightly agree”) and “disagreement” (“slightly disagree”, “disagree”, and “strongly disagree”). In the intervention group, the greatest degree of agreement was observed for the statements “OSCEs should be implemented in the clinical pharmacy course” (100% agreement), “the OSCE seminar imparted practical skills” (100% agreement), and “the OSCE seminar improved clinical skills” (100% agreement). In the control group, the greatest degree of agreement was observed for the statement “the OSCE seminar imparted practical skills” (75% agreement). No participant from either group agreed with the statement “OSCEs are unnecessary because nothing wrong can be done during patient counseling” (0% agreement).

### 3.7. Preparation Questionnaire

The proportions of participants who prepared themselves for the summative OSCEs, the duration of preparation, and the tools used for preparation are shown in Table 6.

## 4. Discussion

This randomized controlled study indicated that our OSCE training approach (using formative OSCEs) was more effective than our university’s non-OSCE training method for improving German pharmacy students in diabetes mellitus counseling. The OSCE training approach (intervention group) showed a significantly greater improvement in counseling and communication skills compared to our university’s traditional approach (control group). Furthermore, the OSCE training approach resulted in a significantly greater increase in self-confidence than the control group’s training.

Our results support the application of our OSCE training approach to improve pharmacy students’ counseling and communication skills. In line with our findings, Gums et al. found that pharmacy students’ communications skills and clinical competency at an ophthalmic OSCE station, as measured by OSCE scores, improved after undergoing individualized formative assessments in a pharmacy skills laboratory [25] which could be considered as a formative OSCE-like approach. In contrast, Chisnall et al. found in a study with historical controls that formative OSCEs as a learning tool did not improve the overall pass rate of students. Nevertheless, they indicated that formative OSCEs were associated with improved pass rates in subsequent summative OSCEs for stations that were identical in the formative and summative OSCE. Additionally, they noted improved pass rates for some stations that did not appear in the formative OSCEs [18]. Alkhateeb et al. found in a randomized control investigation with medical students that applying formative OSCEs as a learning tool in addition to a standard module did not result in a significant difference in pass rates and that the group without formative OSCEs achieved an even higher mean score than the intervention group [20]. Differences in the implementation of our OSCE training approach might explain the positive results of our study. For example, our investigation used a more intensive and interactive training setting than Alkhateeb et al. and Chisnall et al. Our training was conducted in groups and incorporated elements of peer-assisted learning, where counseling performances in OSCE cases on diabetes were observed and assessed by peers’ and trainers’ who provided immediate feedback. In contrast, Alkhateeb et al. and Chisnall et al. applied their OSCEs under examination-like conditions and provided delayed feedback [18,20]. In our study, the formative OSCEs and summative OSCEs required the same skill and knowledge—specifically, counseling and communication for diabetes mellitus. On the other hand, Chisnall et al. and Alkhateeb et al. worked on several stations during their formative OSCEs [18,20]. We assume that these differences in the setting of our training OSCEs contributed to our positive results.

For the study at hand, we assume that the difference between the counseling performance of the groups were not due to a difference in knowledge regarding diabetes mellitus, as the majority of both groups achieved similarly high scores on the first multiple-choice test. Surprisingly, both groups scored more poorly on the second multiple-choice test. The questions used in the multiple-choice tests were based on the diabetes mellitus handout and evaluated basic knowledge on diabetes and not counseling skills. Several reasons might explain the deterioration in the scores on the second multiple-choice test. The observed deterioration in test scores could have resulted from information from the diabetes mellitus handout being committed only to short-term memory and the students may not have revised it for the second multiple-choice test 14 days later as intensively as for the first multiple-choice test. Additionally, the students had little room for improvement in scores from the first multiple-choice test (92% and 83.33% in intervention and control groups, respectively, achieved 100%).

We do not believe that participants’ performance in our study was affected by additional professional education. Although a higher proportion of participants in the intervention group was additionally trained as pharmaceutical technician assistants than in the control group, a greater proportion of the control group, currently or formerly, worked in a community pharmacy in a counseling position, potentially balancing these effects. It should be noted that a higher proportion of participants in the intervention group reported preparing for post-training OSCEs than the control group which may also affect the participants’ OSCE performance. However, not all participants provided information about their preparation and recall bias regarding their preparation were possible.

We sought to optimize measurements for our particular study. For example, the analytical checklist applied to measure participants’ performance during the OSCEs was modified from the PharmAdhere study [36]. Unlike the PharmAdhere study, our analytical checklist did not have weighted items. The weighting of checklist items allows for their differential contribution to the overall score and emphasizes particular items [39,40]. Sandilands et al. did not find “appreciable differences in reliability” by weighting checklists items [40]. Overall, the literature suggests that “the benefit of weighting items is not worth the extra effort” [41]. Based on these counterarguments we decided against weighting. The global rating scale was a shortened version of that from PharmAdhere to facilitate handling for the observers. The self-assessment questionnaire from PharmAdhere was optimized for the use of students and adapted to diabetes content and counseling skills.

Applying the OSCE training approach resulted in a greater increase in self-confidence, as demonstrated by a greater increase in the intervention group’s self-assessment score compared to the control group. This could be expected as OSCE training exposes students to a skill-based educational approach. McClimens et al. revealed a significant increase in confidence once the students have completed the OSCE task [42]. Moreover, a study by Bevan et al. found that practicing OSCEs leads to an improvement of self-confidence [43]. Additional support for implementing OSCEs in training and assessment is the high satisfaction of students in our study. The literature also shows students’ acceptance of OSCEs as an assessment method [44] and as a training approach [18].

### Limitations

We are aware that our research has some limitations. First, standardized patients were portrayed by pharmacists or pharmacy students rather than professional actors. We purposed to overcome this potential bias by providing verbal and written instructions outlining, for instance, the behavior, exemplary dialog, and medical information to the standardized patients. In our study, it was advantageous that the standardized patient was not an observer and could therefore focus on their portrayal.

Second, potential inter-observer bias from the use of three observers instead of one was overcome by maintaining the same observer for each participant between the summative pre- and post-training OSCEs. Additionally, we sought to minimize the possible inter-observer variability by providing examples of correct statements for every item on the analytical checklist and instructions for filling in both the analytical checklist and global rating scale. The use of three observers who had experience in OSCE assessment facilitated the execution of OSCEs with 49 students within a limited timeframe.

Third, the analytical checklist was only exposed to the intervention group during the OSCE training. This could have biased the results of the summative post-training OSCEs as the control group was unaware of the analytical checklist and the criteria for the counseling performance during the OSCEs. However, Cole et al. had found in their controlled study that despite such exposure of scoring rubric in both the intervention and control group there was a significant difference between the groups that indicated the benefit of the peer-led station training (intervention group) [32]—a formative OSCE-like approach. Thus, it might be speculated that the knowledge of the analytical checklist has not substantially affected the performance of the intervention group compared to the control group in our study.

## 5. Conclusions

Counseling patients on medications is one of the key tasks of community pharmacists. As the majority of pharmacists work in community pharmacies [4], it is vital to prepare pharmacy students appropriately to provide adequate counseling right from the beginning of their working life. This study demonstrated that our OSCE training approach provides effective training of counseling and communication skills in the field of diabetes mellitus in a safe environment without jeopardizing patients. These results recommend the widespread use of such a skill-based educational approach in the pharmacy curriculum.

## Figures and Tables

**Table 1:** Demographic characteristics.

	Intervention Group (n = 25)	Control Group (n = 24)
**Age in years**		
Mean (SD)	26.20 (± 6.14)	24.96 (± 5.80)
Median	25	23.5
Range (minimum to maximum)	21–49	20–50
**Gender**		
Female, n (%)	18 (72)	16 (66.67)
Male, n (%)	7 (28)	8 (33.33)
**Experience**		
Additional education as pharmaceutical technician assistant Yes, n (%)	5 (20)	1 (4.17)
Currently or formerly working in a community pharmacy (counseling patients) Yes, n (%)	2 (8)	6 (25)

**Table 2:** Scores achieved by the intervention and control group in the analytical checklist are stated for each section.

Group	Summative Pre-Training OSCE Score in %		Summative Post-Training OSCE Score in %		Score Difference in Percentage Points	
	Mean (SD)	Median (IQR)	Mean (SD)	Median (IQR)	Mean (SD)	Median (IQR)
**Section 1 Greeting**						
**Intervention**	4 (11.06)	0 (0)	88 (18.95)	100 (33.33)	84 (19.53)	100 (33.33)
**Control**	5.56 (12.69)	0 (0)	13.89 (19.45)	0 (33.33)	8.33 (20.26)	0 (16.67)
**Section 2 Medical history**						
**Intervention**	37.26 (14.60)	40 (14.29)	72.34 (25.83)	80 (42.86)	35.09 (28.79)	37.14 (40)
**Control**	47.74 (16.80)	42.86 (20)	53.10 (16.74)	57.14 (20)	5.36 (16.19)	2.86 (20)
**Section 3.1/3.2 Information on the drug (Initiation/Implementation)**						
**Intervention**	33.42 (26.31)	33.33 (37.5)	71.90 (24.30)	75 (30.36)	38.48 (36.28)	44.64 (35.71)
**Control**	38.84 (17.73)	35.42 (26.79)	35.49 (31.41)	35.42 (53.57)	−3.35 (33.53)	−7.44 (43.81)
**Section 3.3 Prevention**						
**Intervention**	27.2 (28.21)	20 (40)	51.2 (22.42)	60 (20)	24 (34.64)	20 (60)
**Control**	14.17 (19.98)	0 (40)	25 (23.77)	20 (40)	10.83 (30.63)	0 (50)
**Section 4 Goal setting**						
**Intervention**	12 (33.17)	0 (0)	32 (47.61)	0 (100)	20 (40.82)	0 (0)
**Control**	16.67 (38.07)	0 (0)	16.67 (38.07)	0 (0)	−8.88 × 10^16^ (58.98)	0 (0)
**Section 5 Patient involvement**						
**Intervention**	46 (49.83)	0 (100)	74 (35.71)	100 (50)	28 (72.28)	50 (100)
**Control**	50 (51.08)	50 (100)	50 (46.63)	50 (100)	−7.03 × 10^16^ (46.63)	0 (0)
**Section 6 Risk-Communication**						
**Intervention**	4 (20)	0 (0)	64 (48.99)	100 (100)	60 (50)	100 (100)
**Control**	0 (0)	0 (0)	20.83 (41.49)	0 (0)	20.83 (41.49)	0 (0)

In each section, different numbers of items were available, and therefore different maximum scores in points were achievable. (SD = standard deviation, IQR = Interquartile range).

**Table 3:** Scores achieved by the intervention and control group in the global rating scale stated for each item.

Group	Summative Pre-Training OSCE Score in %		Summative Post-Training OSCE Score in %		Score Difference in Percentage Points	
	Mean (SD)	Median (IQR)	Mean (SD)	Median (IQR)	Mean (SD)	Median (IQR)
**Verbal communication skills**						
**Intervention**	51.2 (23.15)	60 (20)	77.6 (15.62)	80 (20)	26.4 (25.64)	20 (20)
**Control**	63.33 (24.79)	60 (30)	68.33 (16.59)	60 (20)	4.8 (21.04)	0 (20)
**Non-verbal communication skills**						
**Intervention**	57.6 (17.63)	60 (0)	76 (15.28)	80 (20)	18.4 (26.41)	20 (40)
**Control**	64.17 (18.63)	60 (20)	70.83 (16.66)	70 (20)	6.4 (18)	0 (20)
**Patient-centered communication skills**						
**Intervention**	53.6 (24.98)	60 (40)	72.8 (19.04)	80 (20)	19.2 (29.71)	20 (40)
**Control**	59.17 (23.94)	60 (40)	66.67 (20.99)	60 (20)	7.2 (27.01)	0 (20)

For each item minimum of 0 and maximum of 5 points were achievable. (SD = standard deviation, IQR = Interquartile range).

**Table 4:** Proportions of participants achieved 0, 1, 2, 3, or 4 points in the multiple-choice tests on diabetes mellitus.

	First Multiple-Choice Test on Diabetes					Second Multiple-Choice Test on Diabetes				
**Score achieved in points**	0	1	2	3	4	0	1	2	3	4
**Proportion of the intervention group in %**	0	0	0	8	92	0	16	4	32.00	48.00
**Proportion of the control group in %**	0	0	0	16.67	83.33	0	4.17	8.33	33.33	54.17

N = 25 in the intervention group and n = 24 in the control group.

**Table 5:** Examples of comments from free-text items of the satisfaction survey.

Group	Free-Text Item	Themes ^1^
**Intervention group**	I particularly liked the following at the OSCE seminar:	• the practical exercise of counseling (simulation) and the practical training intervention
		• the practical relevance
		• the checklist as a good guide
	I would change the following:	• time schedules for the OSCEs
		• checklist or OSCEs were not realistic
		• all students should have the possibility to complete the OSCE training intervention
**Control group**	I particularly liked the following at the OSCE seminar:	• the practical exercise of counseling (simulation)
		• the practical relevance
		• feedback
	I would change the following:	• the intervention group was better trained for the counseling was trained better, while preparing SOAP notes in the control group did not prepare for patient counseling
		• provision of guidance document (checklist)
		• several topics should be covered

^1^ The three most frequent themes of comments for each item per group are shown. If themes of comments occurred with equal frequency, one of them was selected.

**Table 6:** Participants’ preparation for the summative OSCEs.

	Intervention Group N ^1^ = 25		Control Group N ^1^ = 24	
	First Summative OSCE	Second Summative OSCE	First Summative OSCE	Second Summative OSCE
**Preparation of themselves**	N = 25	N = 25	N = 24	N = 24
Yes	92.00%	84.00%	91.67%	50%
No	8.00%	8.00%	8.33%	29.17%
n.a.	0.00%	8.00%	0%	20.83%
**Duration of preparation**	N = 23	N = 21	N = 22	N = 12
≤30 min	26.09%	61.90%	45.45%	66.67%
>30 min–≤1 h	30.43%	19.05%	22.73%	16.67%
>1 h–≤2 h	30.43%	19.05%	13.64%	16.67%
>2 h–≤3 h	13.04%	0.00%	18.18%	0.00%
>3 h	0.00%	0.00%	0.00%	0.00%
**Tool used for the preparation**	N = 23	N = 21	N = 22	N = 12
Handout	100.00%	66.67%	100%	83.33%
Textbooks	4.35%	4.76%	0.00%	0.00%
Internet	8.70%	0.00%	9.09%	8.33%
Own notes for other seminars	4.35%	0.00%	9.09%	0.00%
Notes from training	-	71.43%	-	16.67%

^1^ Not all participants filled out the survey entirely. Therefore, the total number of responses differed depending on the item.

## Supplementary Materials

The following are available online at https://www.mdpi.com/2226-4787/8/4/229/s1, Supplemental Material 1: OSCE checklist case 1, Supplemental Material 2: OSCE checklist case 2, Supplemental Material 3: Multiple-choice test 1, Supplemental Material 4: Multiple-choice test 2, Supplemental Material 5: Self-assessment questionnaire.

## Appendix A

**Table A1:** Short description of OSCE (Objective Structured Clinical Examination) cases.

OSCE Case	Short Case Description	Number of Items in the Case
**Summative pre-training OSCE**		
Case 1	Implementation of metformin treatment The standardized patient hands in a prescription on metformin. The participant needs to find out that the prescription is a follow-up prescription and counsels according to this information. Questions the standardized patient asks if the participant asks for open questions (see analytical checklists Section 5 item 5.1): ⚬ Next week, I have a medical appointment for a CT scan for the examination of my arteries. The nurse said that I would then be injected with iodine-based contrast media. I forgot to tell the nurse that I also take the metformin because I get it from another doctor. Do I have to pay attention to something?”	22
Case 2	Initiation of dapagliflozin treatment The standardized patient hands in a prescription on dapagliflozin. The participant needs to find out that the prescription is a first-time prescription and counsels according to this information. Questions the standardized patient asks if the participant asks for open questions (see analytical checklists Section 5 item 5.1): ⚬ “I have read that some medications for the treatment of diabetes make you fat and now I am paying special attention to my diet. Is this the case with this drug?”	27
Case 3	Implementation of sitagliptin treatment The standardized patient hands in a prescription on sitagliptin. The participant needs to find out that the prescription is a follow-up prescription and counsels according to this information. A problem occurred during therapy reported by the patient if the participant asks for adverse drug reactions and interactions that have occurred (see analytical checklists Section 3.2 item 3.2.9) ⚬ “I’ve had hypoglycemia lately, although I haven’t changed my diet.”	22
Case 4	Initiation of acarbose treatment The standardized patient hands in a prescription on acarbose. The participant needs to find out that the prescription is a first-time prescription and counsels according to this information.	24
Case 5	Implementation of glibenclamide treatment The standardized patient hands in a prescription on glibenclamide. The participant needs to find out that the prescription is a follow-up prescription and counsels according to this information. A problem occurred during therapy reported by the patient if the participant asks for adverse drug reactions and interactions that have occurred (see analytical checklists Section 3.2 item 3.2.9) ⚬ “I had low blood sugar a short time ago when I took the drug.” Questions the standardized patient asks if the participant asks for open questions (see analytical checklists Section 5 item 5.1): ⚬ “I would like to buy a pack of plasters. I have a big wound on the foot that has not healed for weeks. Can you recommend something good?”	23
Case 6	Initiation of insulin glargine treatment The standardized patient hands in a prescription on insulin glargine. The participant needs to find out that the prescription is a first-time prescription and counsels according to this information. Questions the standardized patient asks if the participant asks for open questions (see analytical checklists Section 5 item 5.1): ⚬ “The doctor said I had to throw the needle away after each injection and use a new needle the next time. Is it really necessary? This would be expensive permanently.”	26
**Summative post-training OSCE**		
Case 7	Initiation of sitagliptin treatment The standardized patient hands in a prescription on sitagliptin. The participant needs to find out that the prescription is a first-time prescription and counsels according to this information. Questions the standardized patient asks if the participant asks for open questions (see analytical checklists Section 5 item 5.1): ⚬ “Sometimes I have a tingling in my feet and hands. Like little ants crawling on my feet and hands. What can I do about it?”	27
Case 8	Implementation of acarbose treatment The standardized patient hands in a prescription on acarbose. The participant needs to find out that the prescription is a follow-up prescription and counsels according to this information. A problem occurred during therapy reported by the patient if the participant asks for adverse drug reactions and interactions that have occurred (see analytical checklists Section 3.2 item 3.2.9): ⚬ “I had low blood sugar a short time ago when I took the drug.” Questions the standardized patient asks if the participant asks for open questions (see analytical checklists Section 5 item 5.1): ⚬ “Does this remedy lead to weight gain?”	24
Case 9	Initiation of glibenclamide treatment The standardized patient hands in a prescription on glibenclamide. The participant needs to find out that the prescription is a first-time prescription and counsels according to this information. Questions the standardized patient asks if the participant asks for open questions (see analytical checklists Section 5 item 5.1): ⚬ “Does this drug cause as many gastrointestinal complaints as metformin?”	25
Case 10	Implementation of insulin lispro treatment The standardized patient hands in a prescription on insulin lispro. The participant needs to find out that the prescription is a follow-up prescription and counsels according to this information. A problem occurred during therapy reported by the patient if the participant asks for adverse drug reactions and interactions that have occurred (see analytical checklists Section 3.2 item 3.2.9): ⚬ “I don’t know if that’s related to the therapy, but I’ve recently noticed that I’ve got such a weird hard lump on my stomach—where I always inject my insulin.” Questions the standardized patient asks if the participant asks for open questions (see analytical checklists Section 5 item 5.1): ⚬ “I have an insulin lispro pen left over. I have forgotten it in the car once. And now it’s cloudy. Can I still use it?”	25
Case 11	Initiation of metformin treatment The standardized patient hands in a prescription on metformin. The participant needs to find out that the prescription is a first-time prescription and counsels according to this information.	25
Case 12	Implementation of glimepiride treatment The standardized patient hands in a prescription on glimepiride. The participant needs to find out that the prescription is a follow-up prescription and counsels according to this information. A problem occurred during therapy reported by the patient if the participant asks for adverse drug reactions and interactions that have occurred (see analytical checklists Section 3.2 item 3.2.9) ⚬ “I had low blood sugar a short time ago when I took the drug.”	23

## Appendix B

**Table A2:** Short description of patient cases used for the training in the control group. Based on the case description the participants solved the patient case by preparing SOAP (Subjective, Objective, Assessment, Plan) notes.

Patient Case	Short Case Description
Case 1	Implementation of pre-mixed insulin The patient with type 1 diabetes mellitus redeems a follow-up prescription with pre-mixed insulin (aspart and protamine). He is in conventional insulin therapy. He complains that the therapy is restrictive because he now has to pay attention to what he eats, when he eats, that he eats, etc. He often suffers from hypoglycemia. He measures his blood glucose 4 times a day. Twice a week he drinks large amounts of alcohol. On these days he has high blood glucose levels before sleep while the levels are very low the next morning. Three times a week he goes to the gym and does excessive training and has hypoglycemia before lunch on these days. He tells that it seems wasteful to use a new needle after each injection and asks if it would be possible to use injection needles several times.
Case 2	Initiation of pioglitazone and implementation of glibenclamide The patient with type 2 diabetes redeems a first-time prescription for pioglitazone. He reports that he has been treated with glibenclamide for several years, but his long-term blood sugar has deteriorated significantly. He had high blood sugar, especially in the morning. He also reports that a few days ago he had to see his internist due to edema and he prescribed furosemide for him. He is a smoker and does not drink alcohol. He also asks for a recommendation for good plasters, as he has had an open wound on his foot for weeks, which does not heal.
Case 3	Implementation of insulin lispro and insulin detemir The patient with diabetes mellitus redeems a follow-up prescription for insulin lispro and insulin detemir and a follow-up prescription for Carvedilol 25 mg tablets. The patient has received Carvedilol for a few months due to increased blood pressure. The patient has experienced sudden hypoglycemic events. The patient has recently switched from human insulin and NPH insulin to insulin lispro and insulin detemir.
Case 4	Implementation of dapagliflozin and metformin The patient with type 2 diabetes mellitus redeems a follow-up prescription for dapagliflozin 10 mg. Dapagliflozin was prescribed in addition to his other medications due to his high blood sugar after meals. He asks for an effective remedy for cystitis due to episodes of burning during urination. He was diagnosed with hypertension. Next week he has an appointment for a CT scan for the examination of his arteries. The nurse explained to him that he would be injected with iodine-based contrast. He forgot to inform the nurse about his actual medication prescribed by his diabetologist. Now he wants to know if there is something he needs to consider. The patient’s actual medications: Metformin 850 mg, Dapagliflozin 10 mg, Candesartan 8 mg, Paracetamol
Case 5	Implementation of sitagliptin The patient with type 2 diabetes mellitus redeems a follow-up prescription for sitagliptin 100 mg. The patient has experienced several hypoglycemic events and asks for a glucose gel. He claims of having inadequate control of blood sugar levels. In the past, he had taken other medicines for the treatment of his diabetes and could not tolerate them. He was diagnosed with hypertension. The patient’s former and actual medication: Glibenclamide 3.5 mg Acarbose 50 mg (discontinued a few months ago due to gastrointestinal intolerance), Metformin 500 mg (discontinued due to intolerance), Valsartan 80 mg, Paracetamol, Atorvastatin 20 mg

## Appendix C

**Table A3:** Scores achieved by the intervention and control group in the analytical checklist, global rating scale, and self-assessment questionnaire.

Group	Summative Pre-Training Score in %		Summative Post-Training Score in %		Score Difference in Percentage Points		*p*-Value ^1^
	Mean (SD)	Median (IQR)	Mean (SD)	Median (IQR)	Mean (SD)	Median (IQR)	
**Analytical checklist**							
**Intervention**	27.16 (12.75)	33.33 (18.18)	68.67 (14.55)	69.57 (27.33)	41.52 (14.63)	43.98 (19.54)	*p* < 0.001
**Control**	31.61 (8.69)	32.58 (13.16)	34.76 (17.64)	30.03 (20.92)	3.16 (14.87)	−0.33 (16.73)	
**Global rating scale**							
**Intervention**	54.13 (18.69)	60 (20)	75.47 (13.84)	80 (13.33)	21.33 (24.42)	20 (26.67)	*p* = 0.007
**Control**	62.22 (19.72)	63.33 (20)	68.61 (14.77)	70 (16.67)	6.39 (17.19)	3.33 (13.33)	
**Self-assessment questionnaire**							
**Intervention**	48.69 (9.20)	48.57 (14.29)	63.66 (11.47)	62.86 (17.14)	14.97 (12.38)	11.43 (17.14)	*p* = 0.001
**Control**	43.81 (12.93)	42.86 (22.86)	48.33 (14.95)	47.14 (24.29)	4.52 (12.03)	2.86 (7.14)	

SD = standard deviation, IQR = Interquartile range. N = 25 in the intervention group and n = 24 in the control group. ^1^ = A one-sided Mann–Whitney test with a significance level of alpha = 0.05 was used to assess the difference between the intervention and the control group.
