# Supplementary material for: Efficacy of an Objective Structured Clinical Examination Training Approach for Training Pharmacy Students in Diabetes Mellitus Counseling: A Randomized Controlled Trial
Source: Pharmacy (Basel). 2020 Nov 26;8(4):229. doi: 10.3390/pharmacy8040229 (PMC7712022; doi:10.3390/pharmacy8040229)
Supplement: Supplementary file 1 [file pharmacy-08-00229-s001.zip › Supplemental Material 1 OSCE Case 1.pdf]

Case 1 – Implementation of metformin

Participant code: ______________________________

Date: ____

Observer: ____________________________________

Actor: ____

Section 1: Greeting: The pharmacist …	Addressed		Comments
**1.1 Introduces himself**<br>*Pharmacist: Hello, my name is …*			
**1.2 Identifies the patient**<br>*Pharmacist: Are you Mrs./ Mr. …? Is the prescription for you?*<br>*Patient: Yes.*			
**1.3 Checks/adds to the patient's record**<br>*Pharmacist: Are you saved in the customer file?*<br>*Patient: No.*			
Section 2: Medical history: The pharmacist …	Addressed	Correct	Comments
**2.1 Reviews the reason for prescription (Rx) / checks self-diagnosis (self-medication)**<br>*Pharmacist: Do you know why your doctor prescribed this drug?*<br>*Patient: I have type 2 diabetes.*			
**2.2 Finds out whether a new drug was prescribed**<br>*Pharmacist: Have you got this prescription for the first time?*<br>*Patient: No, I already know that.*			
**2.4 Asks about any other prescription drugs the patient is taking**<br>*Pharmacist: What other prescription drugs do you take?*<br>*Patient: Atorvastatin 20 mg once a day in the evening for my high cholesterol levels and enalapril 20 mg once a day in the morning for my high blood pressure.*			
**2.5 Asks about any self-medication products, phytotherapeutics, etc. the patient is taking**<br>*Pharmacist: What other drugs do you take? For example, non-prescription drugs, herbal drugs, or medicines from the drugstore.*<br>*Patient: Sometimes paracetamol when I have a headache.*			
**2.6 Asks about other medical conditions (i.e. chronic diseases, allergies, pregnancy/lactation, kidney/liver impairment)**<br>*Pharmacist: Is there anything else that I should know about your health to give you the best possible advice?*<br>*Patient: What do you mean?*<br>*Pharmacist: For instance, other chronic diseases, allergies, pregnancy, breastfeeding period, kidney or liver impairment?*<br>*Patient: I have diabetes, high blood pressure, and high cholesterol. Nothing else.*			
Section 3.2: Implementation of the therapy: The pharmacist…	Addressed	Correct	Comments
**3.2.1 Asks about any benefits the patient experienced when taking the medication**<br>*Pharmacist: How has your therapy changed your blood sugar levels?*<br>*Patient: My blood sugar levels have improved compared to the last examination.*			
**3.2.2 Asks the patient how often he took the medication**<br>*Pharmacist: How often do you take the medicine?*<br>*Patient: I take metformin (500 mg) 3 times a day.*			

**3.2.3 Identifies how the patient took the medication**<br>*Pharmacist: How/ when do you take the medicine?*<br>*Patient: I always take the drug during meals.*			
**3.2.6 Checks that appropriate monitoring has occurred**<br>*Pharmacist: Do you keep a blood sugar diary?*<br>*Patient: Yes, whenever the doctor has measured my blood sugar levels, I have it entered.*<br>*Or*<br>*Pharmacist: When was the last time your kidney function was checked?*<br>*Patients: About 2 months ago. Everything was fine there.*			
**3.2.9 Identifies any adverse drug reactions that were experienced**<br>*Pharmacist: Have problems or side effects occurred with your therapy? Or Did you have any problems with your medicine?*<br>*Patient: No.*			
Section 3.3: Preventive measures: The pharmacist talks about …	Addressed	Correct	Comments
**3.3.1 Smoking cessation**<br>*Pharmacist: Do you smoke?*<br>*Patient: Yes.*<br>*Pharmacist: It is generally known that smoking is not healthy. This is especially true for patients with diabetes. Smoking increases the risk of diabetic complications and lung diseases. Can you imagine stop smoking? We can also arrange an appointment where we can discuss smoking cessation.*<br>*Patient: Patient: All right. I will contact you for an appointment.*			
**3.3.2 Obesity**<br>*Pharmacist: Weight control also plays a major role, which has a positive impact on the course of the disease.*			
**3.3.3 Physical exercise**<br>*Pharmacist: Physical activity has also a positive impact on your health status. You should do this as regularly as possible, if possible several times a week.*			
**3.3.4 Healthy diet**<br>*Pharmacist: When it comes to nutrition, it is important that you eat a well-balanced diet, e.g. you should prefer vegetable fats and eat high fiber foods.*			
3.3.5 Alcohol consumption<br>*Pharmacist: The consumption of alcohol is problematic. Alcohol can cause low blood sugar levels. Especially during treatment with metformin alcohol consumption should be avoided. Do you drink alcohol?*<br>*Patient: I do not drink alcohol.*			
Section 4: Goal setting: The pharmacist …	Addressed		Comments
**4.1 Sets individual goals**<br>*Pharmacist: Could you be so kind and summarize how you will proceed?*<br>*Patient: I will take my medication as before and contact you about an appointment regarding smoking cessation.*			
Section 5: Patient involvement: The pharmacist …	Addressed	Correct	Comments
**5.1 Invites the patient to ask questions**<br>*Pharmacist: Do you have any questions?*<br>*Patient: Yes, next week, I have a medical appointment for a CT scan for the examination of my arteries. The nurse said that I would then be injected with iodine-based contrast media. I forgot to tell the nurse that I also take the metformin because I get it from another doctor. Do I have to pay attention to something?*			

**5.2 Takes patient's questions into account during the consultation**<br>*Pharmacist: Yes, you have to stop taking metformin before the iodine-based contrast media is applied and you can continue taking metformin at the earliest 48 hours after the administration of the iodine-based contrast media if the control of the kidney function showed stable values. It is important to inform your doctor that you are taking metformin before your appointment.*			
Section 6: Risk communication: The pharmacist …	Addressed		Comments
**6.2 Advises the patient to consult with their physician or pharmacist if questions arise/Suggests a new appointment**<br>*Pharmacist: If any question arises in the meantime, you are welcome to contact your doctor or us at any time.*<br>*Patient: Thank you.*			
Sum			
