# Supplementary material for: Efficacy of an Objective Structured Clinical Examination Training Approach for Training Pharmacy Students in Diabetes Mellitus Counseling: A Randomized Controlled Trial
Source: Pharmacy (Basel). 2020 Nov 26;8(4):229. doi: 10.3390/pharmacy8040229 (PMC7712022; doi:10.3390/pharmacy8040229)
Supplement: Supplementary file 1 [file pharmacy-08-00229-s001.zip › Supplemental Material 2 OSCE Case 2.pdf]

Case 2 – Initiation dapagliflozin

Participant code: ______________________________

Date: ____

Observer: _____________________________________

Actor: ____

Section 1: Greeting: The pharmacist …	Addressed		Comments
**1.1 Introduces himself**<br>*Pharmacist: <u>Hello, my name is …</u>*			
**1.2 Identifies the patient**<br>*Pharmacist: <u>Are you Mrs./ Mr. …? Is the prescription for you?</u>*<br>*Patient: Yes.*			
**1.3 Checks/adds to the patient's record**<br>*Pharmacist: Are you saved in the <u>customer file</u>?*<br>*Patient: No.*			
Section 2: Medical history: The pharmacist …	Addressed	Correct	Comments
**2.1 Reviews the reason for prescription (Rx) / checks self-diagnosis (self-medication)**<br>*Pharmacist: <u>Do you know why your doctor prescribed this drug?</u>*<br>*Patient: I have type 2 diabetes.*			
**2.2 Finds out whether a new drug was prescribed**<br>*Pharmacist: <u>Have you got this prescription for the first time?</u>*<br>*Patient: Yes.*			
**2.3 Asks about the information already provided to the patient by the physician**<br>*Pharmacist: <u>What information did the doctor give you about the drug?</u>*<br>*Patient: Patient: Nothing special. Only that my long-term blood sugar is too high. And that is why I should take this new drug now.*			
**2.4 Asks about any other prescription drugs the patient is taking**<br>Pharmacist: <u>What other prescription drugs do you take?</u><br>*Patient: I take metformin 1000 mg 3 times a day and enalapril 20 mg once a day because of my high blood pressure.*			
**2.5 Asks about any self-medication products, phytotherapeutics, etc. the patient is taking**<br>*Pharmacist: <u>What other drugs do you take? For example, non-prescription drugs, herbal drugs, or medicines from the drugstore.</u>*<br>*Patient: I take nothing else.*			
**2.6 Asks about other medical conditions (i.e. chronic diseases, allergies, pregnancy/lactation, kidney/liver impairment)**<br>*Pharmacist: Is there anything else that I should know about your health to give you the best possible advice?*<br>*Patient: What do you mean?*<br>*Pharmacist: For instance, <u>other chronic diseases, allergies, pregnancy, breastfeeding period, kidney or liver impairment?</u>*<br>*Patient: I have diabetes and high blood pressure. Nothing else.*			
**2.7 Tells the patient the name of the active substance**<br>Pharmacist: The doctor has prescribed the active ingredient dapagliflozin 10 mg.			

Case 2 – Initiation dapagliflozin

Section 3.1: Initiation of the therapy: The pharmacist …	Addressed	Correct	Comments
**3.1.1 Explains the benefit of drug therapy**<br>*Pharmacist: Dapagliflozin, causes an increased glucose excretion in the urine and thus it improves your blood glucose level. Better blood glucose control can reduce the risk of consequential damage from diabetes mellitus.*			
**3.1.2 Explains the patient the correct dosage**<br>*Pharmacist: Take the medicine once a day.*			
**3.1.3 Gives specific instructions for the use of the drug (inhalation, spray, injection; ingestion with or without food)**<br>*Pharmacist: You can take the medicine regardless of meals.*			
**3.1.4 Explains the duration of the therapy**<br>*Pharmacist: The treatment with this medicine is a long-term therapy. Please do not stop taking the medicine if not told so by your doctor.*			
**3.1.6 Explains possible monitoring activities associated with the drug therapy**<br>Pharmacist: Do you keep a blood sugar diary?<br>Patient: Yes, whenever the doctor has measured my blood sugar levels, I have it entered.			
**3.1.7 Provides information on memo-techniques**<br>*Pharmacist: Can you remember the dosage? Or should I write it down for you?*<br>*Patient: Thank you, I can remember.*			
**3.1.8 Tells the patient about potential adverse drug reactions that may occur**<br>*Pharmacist: The mechanism of action of this medicine is the excretion of sugar in the urine. However, this can favor urinary tract infections.*			
**3.1.10 Tells the patient how to manage any adverse drug reactions**<br>*Pharmacist: If this side effect occurs, please see your doctor.*			
Section 3.3: Preventive measures: The pharmacist talks about …	Addressed	Correct	Comments
**3.3.1 Smoking cessation**<br>*Pharmacist: Do you smoke?*<br>*Patient: No.*			
**3.3.2 Obesity**<br>*Pharmacist: Weight control also plays a major role, which has a positive impact on the course of the disease.*			
**3.3.3 Physical exercise**<br>*Pharmacist: Physical activity has also a positive impact on your health status. You should do this as regularly as possible, if possible several times a week.*			
**3.3.4 Healthy diet**<br>*Pharmacist: When it comes to nutrition, it is important that you eat a well-balanced diet, e.g. you should prefer vegetable fats and eat high fiber foods.*			
3.3.5 Alcohol consumption<br>*Pharmacist: The consumption of alcohol is problematic. Alcohol can cause low blood sugar levels. Especially during treatment with metformin alcohol consumption should be avoided. Do you drink alcohol?*<br>*Patient: I do not drink alcohol.*			
Section 4: Goal setting: The pharmacist …	Addressed		Comments
**4.1 Sets individual goals**<br>*Pharmacist: Could you be so kind and summarize how you will proceed?*<br>*Patient: I will take this new medicine once a day and if I get a urinary tract infection I go to the doctor.*			
Section 5: Patient involvement: The pharmacist …	Addressed	Correct	Comments

**5.1 Invites the patient to ask questions**<br>*Pharmacist: Do you have any questions?*<br>*Patient: I have read that some medications for the treatment of diabetes make you fat and now I am paying special attention to my diet. Is this the case with this drug?*			
**5.2 Takes patient's questions into account during the consultation**<br>*Pharmacist: No. Studies have even shown that dapagliflozin therapy can lead to weight loss.*			
Section 6: Risk communication: The pharmacist …	Addressed		Comments
**6.2 Advises the patient to consult with their physician or pharmacist if questions arise/Suggests a new appointment**<br>*Pharmacist: If any question arises in the meantime, you are welcome to contact your doctor or us at any time.*<br>*Patient: Thank you.*			
Sum			
