# Supplementary material for: Efficacy of an Objective Structured Clinical Examination Training Approach for Training Pharmacy Students in Diabetes Mellitus Counseling: A Randomized Controlled Trial
Source: Pharmacy (Basel). 2020 Nov 26;8(4):229. doi: 10.3390/pharmacy8040229 (PMC7712022; doi:10.3390/pharmacy8040229)
Supplement: Supplementary file 1 [file pharmacy-08-00229-s001.zip › Supplemental Material 3 Multiple-choice test 1.pdf]

Knowledge assessment 1 (Multiple-choice test on diabetes mellitus)

Participant code: _______________________

Date: _________________________________

Please tick the correct answer. **There is always only one answer correct.**

1) Which statement about the HbA1c value is correct?
   (A) In order to prevent complications for patients with type 2 diabetes mellitus, the HbA1c value should be in the range of 6.5 - 7.5%, taking individual therapy goals into account.
   (B) The HbA1c value gives information about hypoglycemia or hyperglycemia that has occurred.
   (C) The HbA1c value is a snapshot and reflects the glucose level of the past few minutes.
   (D) An HbA1c value below 6.5% is a diagnostic criterion for diabetes mellitus.

2) Which statement about hypoglycemia is correct?
   (A) Ideally, hypoglycemia should be treated with fatty foods to accelerate absorption.
   (B) Mild hypoglycemia should not be treated.
   (C) A possible cause of hypoglycemia is forgetting an insulin injection.
   (D) Typical symptoms of hypoglycemia include tremors, sweating, cravings, and dizziness.

3) Which statement about type 2 diabetes mellitus is correct?
   (A) Metformin is the first choice for type 2 diabetes mellitus treatment.
   (B) Patients with type 2 diabetes mellitus should monitor their blood glucose more often than patients with type 1 diabetes mellitus, especially if they are treated with metformin.
   (C) Type 2 diabetes mellitus usually manifests itself acutely and within a few days.
   (D) Patients with type 2 diabetes mellitus do not experience hypoglycemia.

4) Which statement about the use of insulin is correct?
   (A) Insulin that is not in use should be stored at 0 ° C if possible.
   (B) Insulin is usually injected into the subcutaneous fat.
   (C) Insulin absorption is slowed down by heat.
   (D) Injection of insulin into the abdomen leads to very slow absorption.
