# Supplementary material for: Efficacy of an Objective Structured Clinical Examination Training Approach for Training Pharmacy Students in Diabetes Mellitus Counseling: A Randomized Controlled Trial
Source: Pharmacy (Basel). 2020 Nov 26;8(4):229. doi: 10.3390/pharmacy8040229 (PMC7712022; doi:10.3390/pharmacy8040229)
Supplement: Supplementary file 1 [file pharmacy-08-00229-s001.zip › Supplemental Material 4 Multiple-choice test 2.pdf]

Knowledge assessment 2 (Multiple-choice test on diabetes mellitus)

Participant code: _______________________

Date: ________________________________

Please tick the correct answer. **There is always only one answer correct.**

1) Which statement about diabetes mellitus is correct?
   (A) The main symptom of diabetes mellitus is hypoglycemia.
   (B) Long-term complications can be micro- and macroangiopathies.
   (C) In most cases, type 1 diabetes mellitus is insulin resistant.
   (D) Type 1 diabetes mellitus usually only manifests in adulthood.

2) Which statement about metformin is correct?
   (A) Metformin is insulinotropic.
   (B) Metformin leads to weight gain.
   (C) Metformin in monotherapy does not lead to hypoglycemia.
   (D) Metformin is the first choice for the treatment of type 1 diabetes mellitus.

3) Which statement about sulfonylureas is correct?
   (A) Sulfonylureas lower insulin secretion.
   (B) A common side effect of sulfonylureas is hyperglycemia.
   (C) Sulfonylureas are not insulinotropic.
   (D) Sulfonylureas can lead to weight gain.

4) Which statement about DPP4-inhibitors is correct?
   (A) SGLT2 inhibitors are insulinotropic.
   (B) Alpha-glucosidase inhibitors reduce postprandial blood sugar spikes.
   (C) DPP4-inhibitors inhibit the action of the incretins.
   (D) Glinides are not insulinotropic.
