# Supplementary material for: Efficacy of an Objective Structured Clinical Examination Training Approach for Training Pharmacy Students in Diabetes Mellitus Counseling: A Randomized Controlled Trial
Source: Pharmacy (Basel). 2020 Nov 26;8(4):229. doi: 10.3390/pharmacy8040229 (PMC7712022; doi:10.3390/pharmacy8040229)
Supplement: Supplementary file 1 [file pharmacy-08-00229-s001.zip › Supplemental Material 5 Self-assessment questionnaire.pdf]

Self-assessment questionnaire

Participant code: ________________________________

Date: ____

How do you assess your competence in the following areas?
Please check the boxes.

---

1. I feel competent in counseling a patient with diabetes mellitus.	☐ Strongly disagree	☐ Disagree	☐ Slightly disagree	☐ Slightly agree	☐ Agree	☐ Strongly agree
2. I feel competent to motivate a patient to carry out the therapy.	☐ Strongly disagree	☐ Disagree	☐ Slightly disagree	☐ Slightly agree	☐ Agree	☐ Strongly agree
3. I feel able to listen actively during a consultation.	☐ Strongly disagree	☐ Disagree	☐ Slightly disagree	☐ Slightly agree	☐ Agree	☐ Strongly agree
4. I feel able to guide the patient through questions.	☐ Strongly disagree	☐ Disagree	☐ Slightly disagree	☐ Slightly agree	☐ Agree	☐ Strongly agree
5. I feel able to structure a consultation based on the time available (10 minutes).	☐ Strongly disagree	☐ Disagree	☐ Slightly disagree	☐ Slightly agree	☐ Agree	☐ Strongly agree
6. I feel competent to pass on correct, relevant, and useful information to the patient during the counseling.	☐ Strongly disagree	☐ Disagree	☐ Slightly disagree	☐ Slightly agree	☐ Agree	☐ Strongly agree
7. I feel competent to convey my specialist knowledge in lay language.	☐ Strongly disagree	☐ Disagree	☐ Slightly disagree	☐ Slightly agree	☐ Agree	☐ Strongly agree
